# Electroconvulsive Therapy Versus Ketamine for Major Depressive Episode

**DOI:** 10.1192/bjo.2026.11243

**Published:** 2026-06-30

**Authors:** James Finlay

**Affiliations:** South Eastern Health and Social Care Trust, Belfast, United Kingdom

## Abstract

**Aims:**

To review and compare the safety, efficacy, cost-effectiveness and remission rates of electroconvulsive therapy (ECT) versus ketamine in patients with major depressive episodes.

**Methods:**

A narrative review of the literature was undertaken, focusing on randomised and non-randomised clinical trials, systematic reviews, and meta-analyses comparing ketamine and ECT in adults with major depressive episodes. Key outcomes studied included depressive symptom severity, suicidal ideation, cognitive effects, safety outcomes, relapse prevention and economic considerations.

**Results:**

Across comparative clinical trials synthesised in recent meta-analyses, ECT demonstrated greater reductions in depressive symptom severity than ketamine across multiple validated outcome measures, including the Montgomery-Åsberg Depression Rating Scale (MADRS), Hamilton Depression Rating Scale (HDRS), and Beck Depression Inventory (BDI). Evidence relating to suicidal ideation was limited, with no clear differences observed between treatments in short-term follow-up. Cognitive outcomes were mixed, with some evidence suggesting better short-term cognitive performance with ketamine, while other studies reported no significant differences. Safety profiles differed between treatments: ECT was associated with headaches and muscle pain, whereas ketamine was more commonly linked to transient dissociative and perceptual symptoms. Cost-utility analyses favoured ECT which generated more quality-adjusted life years at a lower incremental cost compared with esketamine. Failure to respond to ECT was not found to predict failure to respond to ketamine also, highlighting a potential role for patients receiving ketamine who have not responded to ECT.

**Conclusion:**

Current evidence suggests that ECT remains superior to ketamine in overall efficacy, remission, and cost-effectiveness for major depressive episodes. However, ketamine may have a role in selected patients, particularly where rapid antidepressant effects are required. Treatment decisions should be individualised and guided by patient preference, clinical urgency and adverse-effect profiles.

